# Body composition components associated with sex differences in resting metabolic rate among Japanese athletes

**DOI:** 10.3389/fspor.2026.1816324

**Published:** 2026-07-02

**Authors:** Nozomi Miura, Mika Goshozono, Kuniko Moto, Honoka Miyashita, Suguru Torii, Akira Namba, Motoko Taguchi

**Affiliations:** 1 Graduate School of Sport Sciences, Waseda University, Tokorozawa, Japan; 2 Faculty of Sport Sciences, Waseda University, Tokorozawa, Japan; 3 Research Institute for Sports Nutrition, Waseda University, Tokorozawa, Japan; 4 Faculty of Human Nutrition, Seitoku University, Matsudo, Japan; 5 Department of Clinical Genomics, Saitama Medical University, Iruma, Japan

**Keywords:** athletes, body composition, fat-free mass, indirect calorimetry, resting energy expenditure, resting metabolic rate, sex differences, skeletal muscle mass

## Abstract

**Introduction:**

Resting metabolic rate (RMR) represents the largest component of total energy expenditure and is fundamental for estimating daily energy requirements in athletes. Although sex is commonly incorporated into predictive equations for RMR, the physiological determinants underlying sex differences remain incompletely understood. This study aimed to identify the body composition components associated with variation in RMR in male and female athletes and comparing the proportion of explained variance between sexes.

**Methods:**

This cross-sectional study included 74 male and 47 female Japanese athletes competing at the national level. Body composition was assessed using dual-energy x-ray absorptiometry. RMR was measured by indirect calorimetry using the Douglas bag technique under standardized fasting and resting conditions. Associations between RMR and body composition variables were evaluated using multiple regression models.

**Results:**

Male athletes exhibited significantly greater fat-free mass (FFM) and skeletal muscle mass (SMM), and lower fat mass (FM) compared with female athletes. Absolute RMR was significantly higher in male athletes; however, RMR adjusted for FFM did not differ between sexes. Multiple regression analyses identified FFM as the primary predictive variable of RMR in both sexes, with SMM emerging as the significant variable when FFM was subdivided. The proportion of variance in RMR explained by these variables was higher in male athletes than in female athletes.

**Conclusions:**

The significant variables of RMR were either FFM or SMM, and were similar in Japanese male and female athletes, but their contributions differed between sexes. Sex differences in RMR may be partly attributable to differences in body composition and absolute body size. Given the cross-sectional design, these findings should be interpreted as associations rather than causal determinants.

## 1 Introduction

Maintaining an appropriate energy balance can help athletes sustain their physical condition and improve their athletic performance (1). Total energy expenditure is primarily composed of the resting metabolic rate (RMR), activity-induced energy expenditure, and diet-induced thermogenesis (2), among which RMR is a key component for estimating energy requirements of athletes. Although numerous regression equations incorporating sex exist for estimating RMR (3–6), the substantive differences between sexes remain unclear.

Understanding the biological differences in body composition and metabolism is essential for developing optimal dietary strategies for male and female athletes. Sex differences in body composition are well documented in nonathletes, with men generally having larger body size, fat-free mass (FFM), and skeletal muscle mass (SMM), whereas women tend to have higher relative and absolute fat mass (FM) (7–9). These differences persist even in highly trained athletes and are fundamental determinants of physiological function. Male athletes exhibit greater FFM and lower body fat percentages than female athletes, and these differences are considered the primary determinants of sex-based differences in physical performance (10). Furthermore, female athletes possess approximately 70% of the SMM found in male athletes, and their skeletal muscle index has been reported to reach approximately 77% of male athletes at the upper limit (11, 12). These studies suggest that fundamental differences exist in body composition of male and female athletes. RMR is significantly influenced by height, body size, and body composition (13). Previous study has reported that the relationships among RMR, body composition, and sex cannot be fully explained by simple adjustments for body size or FFM alone (14). In addition, prediction accuracy has been shown to differ by sex, and FFM-based approaches tend to provide better estimates of RMR than body weight–based approaches (15). Given the differences in physique and body composition between male and female athletes, it is plausible that RMR varies by sex. It has been reported to be higher in male athletes than in female athletes (3, 4, 16–19). However, several important gaps remain in the current literature. Most previous studies have focused primarily on total body size or FFM (4, 17–19), whereas the contribution of specific FFM compartments, particularly SMM, to sex differences in RMR has not been sufficiently examined in athletes. In addition, studies directly comparing male and female athletes within the same ethnic population are limited. Furthermore, although menstrual cycle phase may influence RMR in women (20), few studies investigating sex differences in athletes have considered menstrual cycle phase during RMR assessment. RMR of athletes is often estimated from the FFM; however, FFM consists of various organ tissues with different metabolic characteristics (21, 22). Therefore, examining body composition at the detailed compartment level may contribute to a more precise understanding of the physiological variables associated with RMR. Absolute RMR is higher in male athletes than in female athletes, primarily because of greater FFM and SMM (17). When the RMR is adjusted for FFM, sex differences are often attenuated or eliminated (16–18), indicating that body composition is a major mediator of sex differences in metabolic rate. Consequently, sex differences in body composition are key physiological variables underlying differences in metabolism, and investigating these differences in athletes may also improve the interpretation of the sex-specific determinants of RMR.

This study aimed to identify the body composition components associated with variation in RMR in Japanese male and female athletes and comparing the proportion of explained variance between sexes. We hypothesized that although absolute body size and body composition differ, the predictive variables for both male and female athletes are FFM or SMM.

## 2 Methods

### 2.1 Study design

This study used a cross-sectional design to compare Japanese male and female athletes. All procedures were approved by the Ethics Review Committee on Research with Human Subjects of Waseda University (application number 2021-219) and adhered to the principles outlined in the Declaration of Helsinki. The participants were recruited between October 2022 and June 2025. Athletes were selected from several sports teams.

### 2.2 Participants

**Figure 1:**
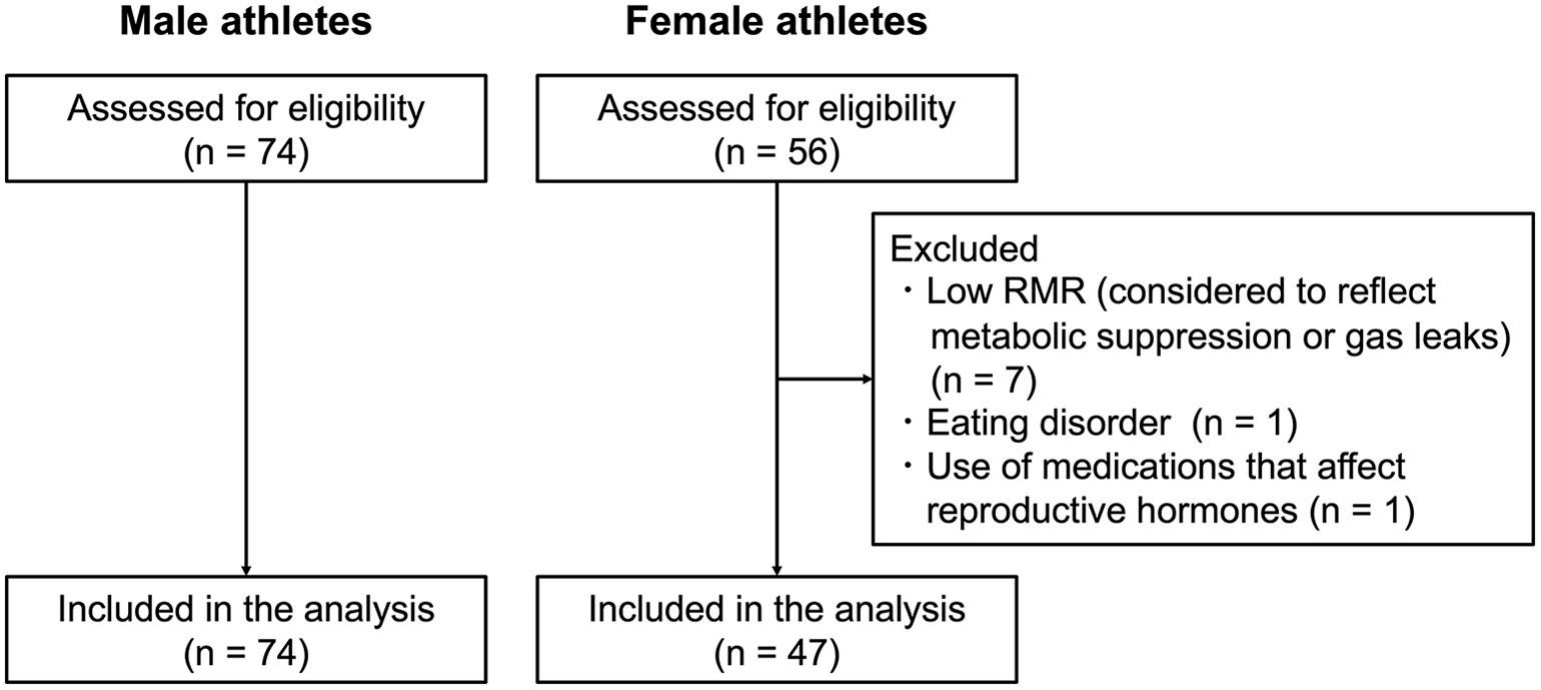
Flowchart demonstrated the participant screening process. RMR, resting metabolic rate.

The participant screening process is illustrated in Figure 1. Initially, 74 male and 56 female athletes expressed interest in the study and agreed to participate. All participants were informed of the study aims and potential risks associated with participation. Written informed consent was obtained from all the participants. The inclusion criteria for both groups were national-level athletes classified as Tier 3 (23). The exclusion criteria for both groups encompassed 1) smoking, 2) use of medications that affect metabolic or reproductive hormones, and 3) major illness or injury. The triiodothyronine (T_3_) levels of all the participants were confirmed to be within the normal range. Measurements were performed during the regular training season for each sport. Weekly training duration and frequency were collected using self-report questionnaires and activity logs. In the female athletes' group, nine individuals were excluded: seven RMR values were identified as outliers (considered to reflect metabolic suppression or to raise concerns about gas leaks due to malfunctioning measuring equipment), one for an eating disorder, and one for the medication use that affects reproductive hormones. Thus, 74 Japanese male athletes (male group; age, 20 ± 1 years) and 47 Japanese female athletes (female group; age, 21 ± 2 years) were included in the analysis. Table 1 shows the distribution of sports (23). The male group comprised individuals participating in long-distance running (*n* = 30), cycling (*n* = 4), short-distance running (*n* = 7), hurdles (*n* = 1), American football (*n* = 17), baseball (*n* = 7), lacrosse (*n* = 7), and auto-racing (*n* = 1). The female group included long-distance running (*n* = 20), Nordic combined (*n* = 3), short-distance running (*n* = 1), lacrosse (*n* = 4), soccer (*n* = 9), rugby (*n* = 5), artistic swimming (*n* = 1), alpine skiing (*n* = 2), ski jumping (*n* = 1), and ice hockey (*n* = 1).

**Table 1:** The distribution of sports.

Sport categories [n (%)]	Male athletes	Female athletes
	(*n* = 74)	(*n* = 47)
Team sports	31 (41.9)	19 (40.4)
Endurance/long distance	31 (41.9)	20 (42.6)
Middle distance/power	3 (4.1)	3 (6.4)
Speed/strength	8 (10.8)	4 (8.5)
Precision/skill-dependent sports	1 (1.4)	1 (2.1)

### 2.3 Body composition

Body weight was recorded to the nearest 0.05 kg using a digital scale (UC-321; A&D Co., Tokyo, Japan) after an overnight fast, whereas height was measured to the nearest 0.1 cm with a stadiometer (YL-65; Yagami, Inc., Tokyo, Japan) with minimal clothing and no shoes.

The body fat percentage, bone mineral content (BMC), and appendicular lean soft tissue mass (ALST) were determined using a dual-energy x-ray absorptiometry (DXA; Horizon A DXA scanner; Hologic Inc., MA, USA). Whole-body images were acquired, and all scans and analyses were performed by a skilled orthopedist using the Hologic software (ver. 12.4.3; Hologic Inc., MA, USA). The mean coefficient of variation (CV) for these measurements was < 1%. FM was derived from body weight and body fat percentage, and FFM was calculated by subtracting FM from body weight. Bone mass (BM) and SMM were estimated using the following prediction models (24, 25):

BM (kg) = BMC (g) × 1.85/1,000
SMM (kg) = 1.13 × ALST (kg) − 0.02 × age (years) + (0.61 × sex: male = 1, female = 0) + 0.97
Residual (kg) = body weight (kg) − FM (kg) − BM (kg) − SMM (kg)

### 2.4 Resting metabolic rate

The RMR was measured by indirect calorimetry using the Douglas bag method. For female athletes, menstrual status was confirmed by self-reported questionnaires before the measurements. All participants reported that they were not amenorrheic and were not using hormonal contraceptives. RMR was measured during the early follicular phase of the menstrual cycle as determined by a gynecologist. Participants were instructed to abstain from caffeine and alcohol for 24 h before the measurement and to avoid strenuous exercise on the day before testing whenever possible. However, complete standardization of training load was not always feasible because all participants were competitive athletes undergoing regular training. At a minimum, all participants refrained from intense physical exercise for at least 10 h before RMR measurement. Participants arrived at the laboratory in the morning after an overnight fast, except for water. Upon arrival, the participants rested in the supine position for at least 30 min to allow their heart rates to reach resting levels, at which point a face mask (Vise Medical, Japan) was fitted. The resting heart rate and body temperature were confirmed to ensure a resting state. The testing environment was maintained at a comfortable temperature (22–24 °C) and kept quiet to minimize disturbances. Throughout the measurements, participants were instructed to remain awake and rested.

The expired gas was collected for 10 min, and sampling was continued until the RMR difference between the two samples was less than 5%. O_2_ and CO_2_ concentrations were analyzed using a gas analyzer (AE-100i; Minato Medical Science Co., Ltd., Osaka, Japan), and expired air volume was measured using a dry gas volume meter (DC-5; Shinagawa, Japan). The Weir equation (26) was used to calculate the final RMR value. The average values of the two samples were used for the analysis. The CV values of male and female athletes were 1.56% for male athletes, 1.75% for female athletes in RMR, 1.49% for male athletes, 2.84% for female athletes in VO_2_, and 2.63% for male athletes, 2.75% for female athletes in VCO_2_, respectively. The mean respiratory quotient was 0.81 (95% confidence interval: 0.80–0.82) for male athletes, 0.82 (95% confidence interval: 0.80–0.83) for female athletes, respectively.

### 2.5 Statistical analysis

Data analysis was conducted using IBM SPSS Statistics (version 29.0.2.0; IBM Corporation, Armonk, NY, USA). The Shapiro–Wilk test was used to assess distributional normality, and nonparametric tests were selected for variables that were not normally distributed (body fat and FM). Between-group differences were examined using unpaired *t*-tests or Mann–Whitney *U*-tests, with effect sizes calculated according to Cohen's *d* (small: *d* = 0.2; medium: *d* = 0.5; large: *d* = 0.8) (27). The relationships among RMR, FFM, and SMM were examined using Pearson's correlation coefficients. Multiple regression analysis with forced entry was used to determine the FM, FFM, SMM, and residual attributes of RMR. Prior to multiple regression analyses, the assumptions of linear regression were assessed, including linearity, normality of residuals, homoscedasticity, and multicollinearity. Residual plots and normal probability plots were visually inspected, and normality was verified using the Shapiro–Wilk test. Multicollinearity was evaluated using variance inflation factors (VIFs) and tolerance values. VIF values < 10 were considered acceptable. All data were reported as mea*n* ± SD. Statistical significance was set at *p* < 0.05.

## 3 Results

All Japanese 74 male and 47 female athletes completed data collection. Participants' characteristics are summarized in Table 2. Male athletes had significantly greater height, body weight, and FFM than female athletes, whereas fat mass and fat percentage were lower in male athletes. Serum T_3_ levels were significantly higher in male athletes than in female athletes (male athletes: 109 ± 15 ng/dL; female athletes: 93 ± 19 ng/dL; *p* < 0.001).

**Table 2:** Characteristics and body composition of the participants.

Characteristics	Male athletes	Female athletes	*p*	Effect size
	(*n* = 74)	(*n* = 47)		
Height (cm)	174.1 ± 5.9	159.8 ± 4.8	<0.001	2.613
Body weight (kg)	67.0 ± 11.8	53.0 ± 6.3	<0.001	1.398
Body fat (%)	11.3 ± 3.1	19.3 ± 3.7	<0.001	2.319
FM (kg)	7.9 ± 3.6	10.3 ± 2.8	<0.001	0.743
FFM (kg)	59.2 ± 8.7	42.7 ± 4.5	<0.001	2.227
BM (kg)	5.0 ± 0.7	3.9 ± 0.5	<0.001	1.703
SMM (kg)	30.7 ± 5.1	20.5 ± 2.4	<0.001	2.375
Residual (kg)	22.1 ± 2.7	18.3 ± 1.9	<0.001	1.874
Training time (h/week)	17.6 ± 5.5	13.5 ± 3.9	<0.001	0.825

Data are reported as mean ± SD. FM, fat mass; FFM, fat-free mass; BM, bone mass; SMM, skeletal muscle mass.

**Figure 2:**
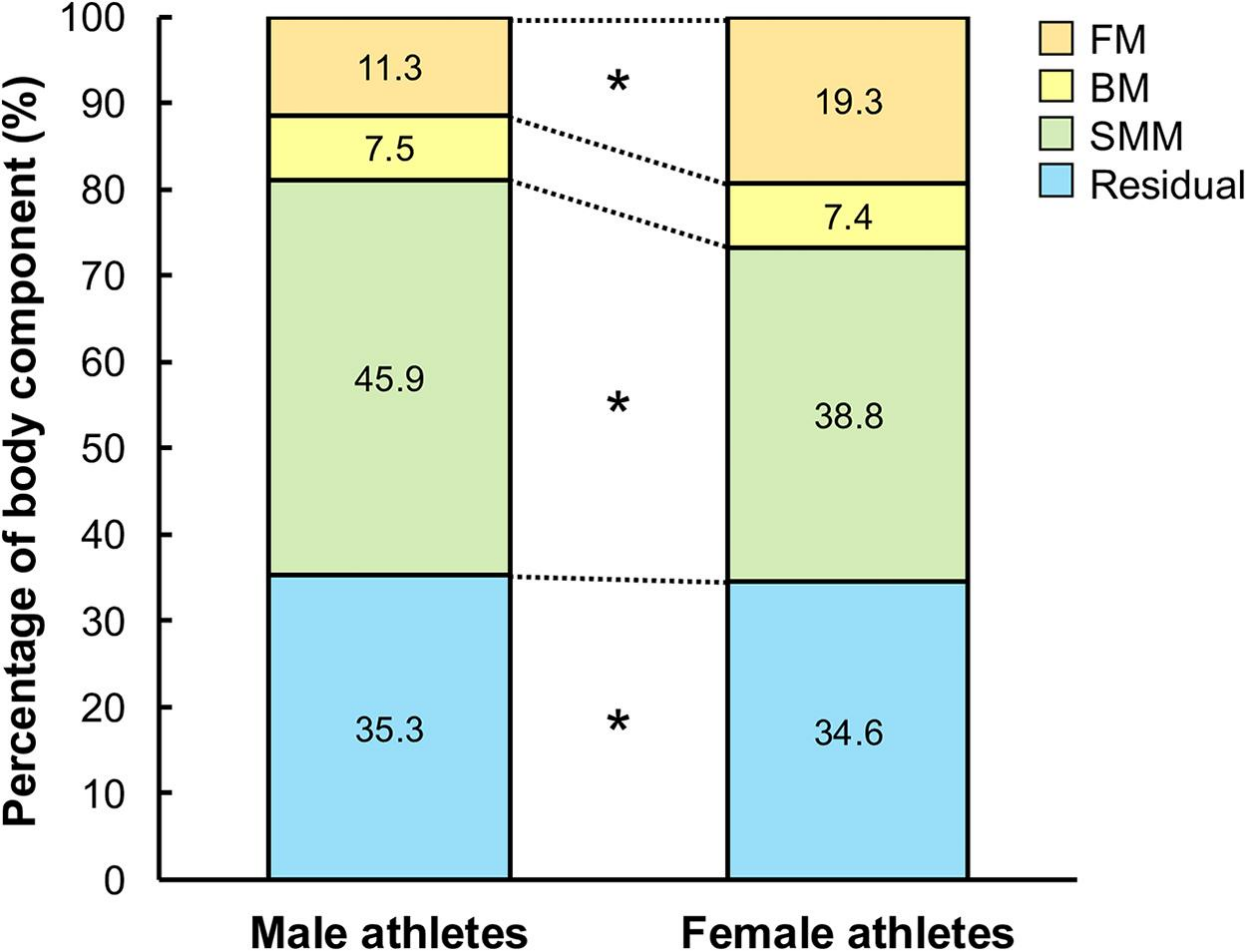
Comparison of the percentage of each body component to body weight. Data were compared using unpaired *t*-tests. *Significant difference between the two groups, *p* < 0.001. FM, fat mass; BM, bone mass; SMM, skeletal muscle mass.

The percentages of each body component are shown in Figure 2. The percentage of the SMM (male athletes: 45.9 ± 1.9%; female athletes: 38.8 ± 2.5%; *p* < 0.001) and residual (male athletes: 35.3 ± 1.9%; female athletes: 34.6 ± 1.9%; *p* = 0.047) was significantly higher in male athletes, whereas the FM percentage was lower in male athletes. However, no significant difference in BM percentage between the groups (male athletes: 7.5 ± 0.8%; female athletes: 7.4 ± 0.7%; *p* = 0.313).

**Figure 3:**
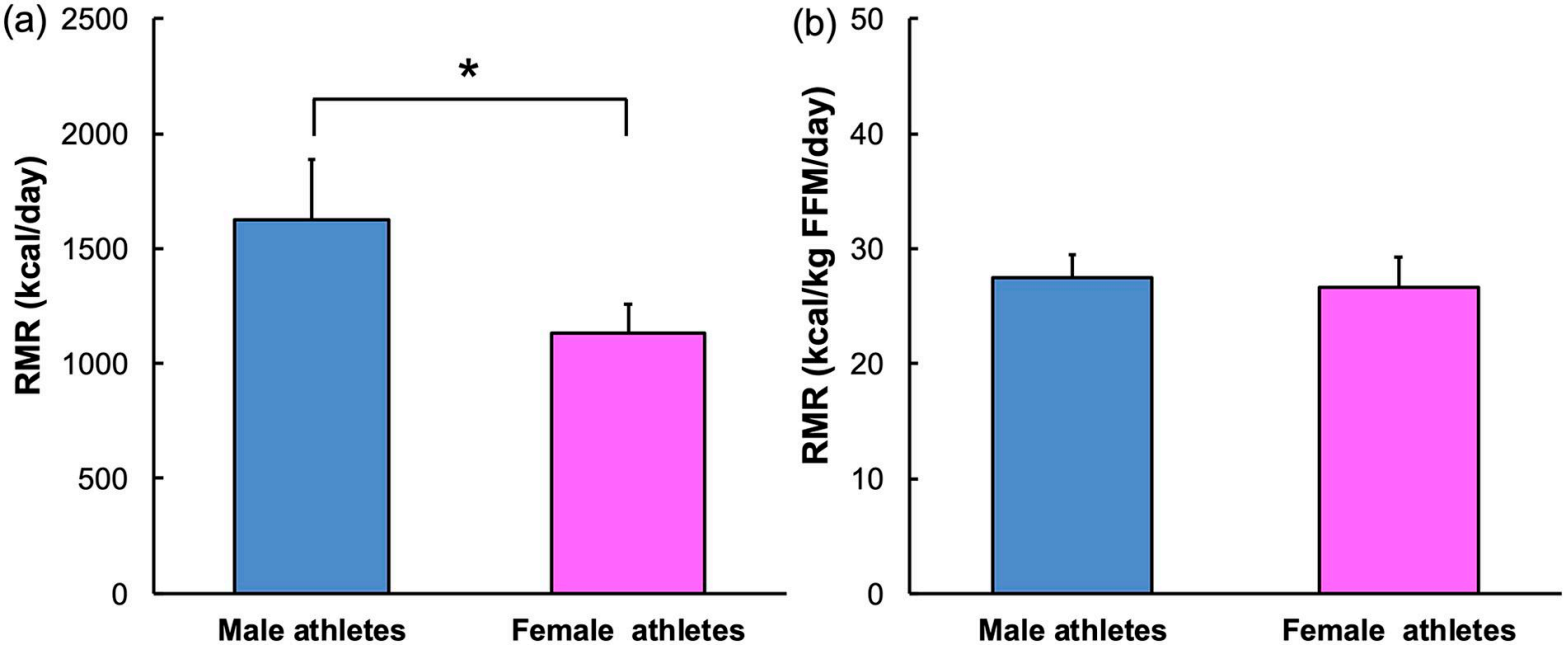
Comparison of RMR. **(a)** Absolute RMR, **(b)** RMR per FFM. Data were compared using unpaired *t*-tests. *Significant difference between the two groups, *p* < 0.001. RMR, resting metabolic rate; FFM, fat-free mass.

The absolute values of RMR and RMR per FFM are shown in Figure 3. Absolute value of RMR was significantly higher in male athletes (male athletes: 1,625 ± 265 kcal; female athletes: 1,135 ± 112 kcal; *p* < 0.001). In contrast, RMR/FFM did not differ significantly between sexes (male athletes: 27.5 ± 2.0 kcal; female athletes: 26.7 ± 2.6 kcal; *p* = 0.060).

**Figure 4:**
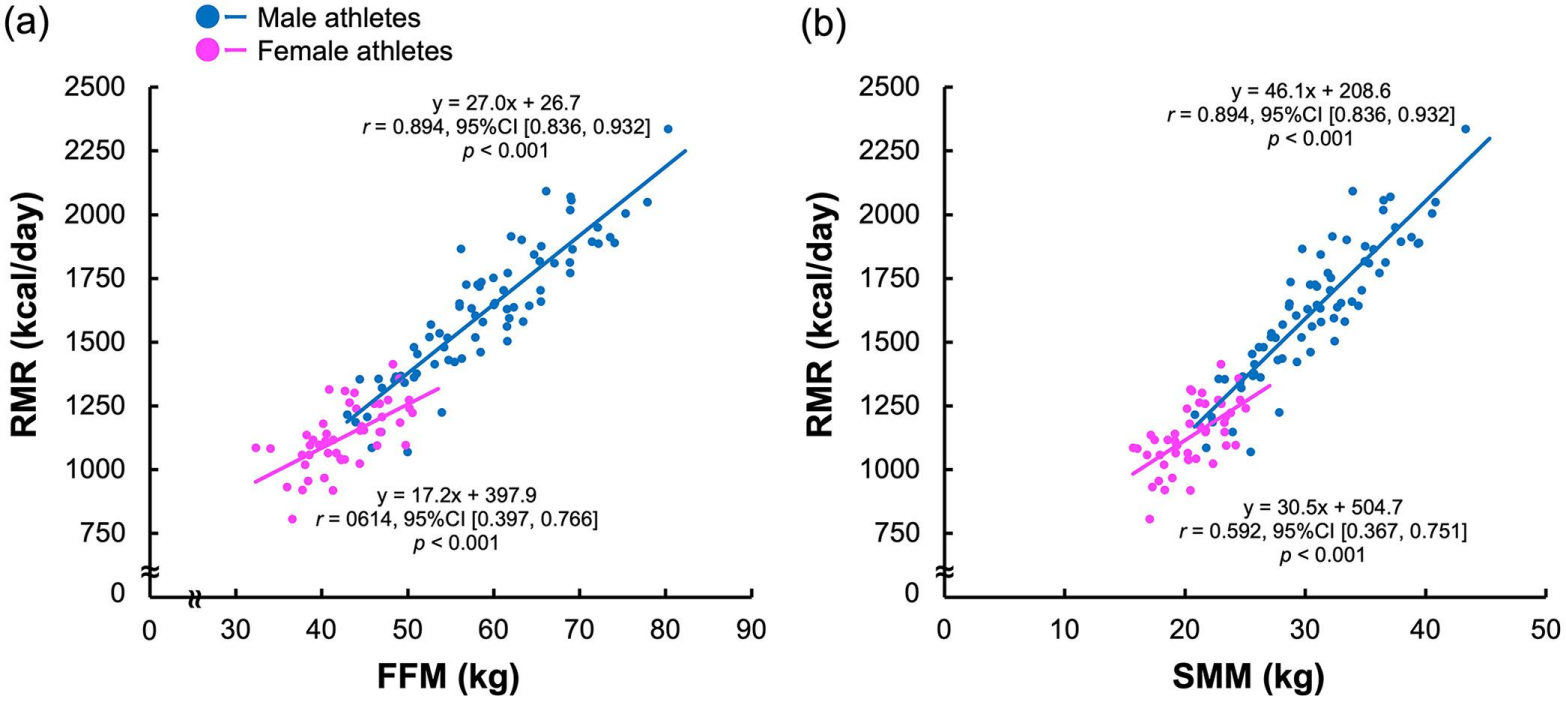
Relationship between RMR and **(a)** FFM, **(b)** SMM. Data were analyzed using Pearson's correlation coefficients. RMR, resting metabolic rate; FFM, fat-free mass; SMM, skeletal muscle mass.

Figure 4 shows the relationship between FFM, SMM, and RMR. Significant positive correlations were observed between RMR and FFM in both male and female athletes [male athletes: *r* = 0.894, 95%CI (0.836, 0.932), *p* < 0.001; female athletes: *r* = 0.614, 95%CI (0.397, 0.766), *p* < 0.001], and between RMR and SMM [male athletes: *r* = 0.894, 95%CI (0.836, 0.932), *p* < 0.001; female athletes: *r* = 0.592, 95%CI (0.367, 0.751), *p* < 0.001]. When integrating data from all athletes, significant positive correlations were also observed between RMR and FFM [*r* = 0.932, 95%CI (0.902, 0.952), *p* < 0.001] and SMM [*r* = 0.931, 95%CI (0.901, 0.952), *p* < 0.001].

 Table 3 shows the effects of the FM, FFM, SMM, and residual on RMR variance. Regression diagnostics indicated no major violations of model assumptions, and residual distributions and homoscedasticity were considered acceptable. Multicollinearity was within acceptable ranges, with all VIF values below 10. The results showed that FFM alone explained 79.4% of the variance in RMR among male athletes and 41.6% among female athletes. In both groups, FM was not an independent variable influencing RMR variance. Model 2, which further subdivided FFM into SMM and residual, explained 80.0% and 42.1% of RMR variance for male and female athletes, respectively. In female athletes, FM approached statistical significance in both regression models, although it did not meet the predefined significance threshold. Confidence intervals for the regression coefficients were relatively wide in female athletes compared with male athletes, indicating greater variability and less precise estimates. Additional analyses adjusted for sport category yielded similar results, with FFM and SMM remaining the primary explanatory variables for RMR. Additional exploratory multiple regression analyses were performed by adding T_3_ to the body composition models. In male athletes, T_3_ was identified as a significant predictor in both Model 1, which included FM, FFM, and T_3_ (R^2^ = 0.810), and Model 2, which included FM, SMM, residual mass, and T_3_ (R^2^ = 0.814). In female athletes, T_3_ was not a significant predictor in either model, while the explanatory power was slightly increased in Model 1 (R^2^ = 0.434) and Model 2 (R^2^ = 0.440). Correlation analyses showed that T_3_ was positively associated with RMR in both male athletes (*r* = 0.368, *p* = 0.001) and female athletes (*r* = 0.377, *p* = 0.010), but the association was weaker than those observed for FFM and SMM. Simple linear regression analysis indicated that T_3_ was significantly associated with RMR in both male athletes (R^2^ = 0.123, *p* = 0.001) and female athletes (R^2^ = 0.142, *p* = 0.010).

**Table 3:** Multiple regression analysis with RMR.

Variables	Male athletes (*n* = 74)							Female athletes (*n* = 47)						
	R^2^	B	SEE	*β*	*t* value	*p*	95%CI	R^2^	B	SEE	*β*	*t* value	*p*	95%CI
Model 1: FM, FFM	0.794							0.416						
FM		−0.48	5.98	−0.01	−0.08	0.936	−12.4, 11.4		10.21	5.95	0.23	1.72	0.093	−1.8, 22.2
FFM		27.17	2.49	0.90	10.92	< 0.001	22.2, 32.1		14.14	3.68	0.51	3.85	< 0.001	6.7, 21.5
Model 2: FM, SMM, Residual	0.800							0.421						
FM		−2.10	6.08	−0.03	−0.35	0.731	−14.2, 10.0		11.87	6.38	0.26	1.86	0.070	−1.0, 24.7
SMM		34.76	7.20	0.67	4.83	< 0.001	20.4, 49.1		22.41	9.58	0.44	2.34	0.024	3.1, 41.7
Residual		21.60	11.99	0.26	1.80	0.076	−2.3, 45.5		4.86	13.62	0.07	0.36	0.723	−22.6, 32.3

RMR, resting metabolic rate; B, partial regression coefficient; SEE, standard error of estimate; β, standardized partial regression coefficient; CI, confidence interval; FM, fat mass; FFM, fat-free mass; SMM, skeletal muscle mass.

## 4 Discussion

The main finding of this study was that for both Japanese male and female athletes, FFM, particularly SMM within FFM, was associated with RMR. While sex differences in RMR per day may be partly explained by differences in body composition and discrepancies in absolute body size, the substantial sex differences were observed in the explanatory power of the regression models. These findings confirm our hypothesis that, although absolute body size and body composition differ between male and female athletes, FFM or SMM would serve as the predictive variables in both sexes; however, a residual sex-related difference remained evident.

Previous studies have shown that male athletes exhibit significantly higher absolute RMR values and greater body weights than female athletes (3, 16–19), and our results are consistent with these findings. Among nonathletes with normal body weight, men have been reported to have a higher RMR than that of women (8, 9, 28, 29). Similarly, the RMR in this study was 1,625 ± 265 and 1,135 ± 112 kcal for male and female athletes, respectively. The absolute value of RMR in male athletes in this study was approximately 200 kcal lower than that in the previous study of Japanese athletes (30, 31), which is likely due to the participants in the previous study having greater body weight (73.9–81.2 kg) and greater FFM (63.0–67.7 kg) than the participants in this study (body weight, 67.0 ± 11.2 kg; FFM, 59.2 ± 8.7 kg). A prior study (32) involving male athletes with similar body weights reported an RMR of 1,643 kcal, consistent with our results. In contrast, the female athletes in this study had body weight, FFM, and RMR similar to those in previous studies (20, 33). We found that the RMR of athletes is determined by body size, as well as in nonathletes (34), and our finding was supported by previous evidence that RMR reflects body size (35). However, the aforementioned sex difference in absolute RMR disappeared in the RMR/FFM, consistent with previous studies (16–18). In this study, the RMR per FFM for male and female athletes was 27.5 ± 2.0 kcal/kg FFM and 26.7 ± 2.6 kcal/kg FFM, respectively. No apparent difference from values reported for Japanese athletes (20, 30, 33), supporting the external validity of our results. Although these findings suggest that sex differences in absolute RMR primarily reflect quantitative differences in FFM, this normalization approach should be interpreted with caution, as simple division by FFM (RMR/FFM) may oversimplify the relationship between body composition and RMR and may not fully account for sex-related differences in body composition and metabolic characteristics.

The FFM is not a single homogeneous entity but is divided into three major components: SMM, BM, and residual. FM and BM have relatively low specific metabolic rates, whereas residuals have high metabolic rates (22). Residuals include various internal organs such as the brain, heart, liver, and kidneys (240, 440, 200, and 440 kcal/kg/day, respectively) (22). These organs exhibit higher organ-specific metabolic rates than other tissues in the body. Although the metabolic rate of the skeletal muscle (13 kcal/kg/day) is lower than that of organs with high metabolic rates included in the residual, athletes have a greater mass of skeletal muscle in the entire body, as shown in Figure 2. The SMM-to-body-weight ratio in this study was 45.9% in male athletes and 38.8% in female athletes, with male athletes exhibiting a significantly higher ratio than female athletes. This value is comparable to those reported in previous studies on Japanese male athletes (approximately 40%–45%) (31, 32, 36, 37) and Japanese female athletes (approximately 40%) (20, 33). On the other hand, the percentage of SMM relative to body weight is lower in nonathletes, at 36%–38% for men and 27%–31% for women (7, 8, 37–39), indicating that a higher SMM is a characteristic of athletes. Muscle hypertrophy generally occurs through exercise (40), and muscle protein synthesis is stimulated by resistance training in athletes (41). The participants of this study were classified as Tier 3 athletes with sports experience since their student days, and it is plausible that their daily training contributed to skeletal muscle hypertrophy. The largest component of FFM in athletes is SMM, which accounted for 51.8% in male athletes and 48.0% in female athletes in this study, yielding nearly identical proportions. SMM accounted for approximately half of the total FFM in both sexes, which may partly explain the absence of sex differences in RMR normalized to FFM. However, sex-related differences in skeletal muscle metabolic characteristics have been reported, including variations in lipid oxidation capacity, mitochondrial density, and muscle fiber type distribution (42, 43), suggesting that the metabolic quality of skeletal muscle may differ between men and women. These elements could influence RMR independently of muscle mass alone. An important methodological consideration is that SMM was not directly measured but estimated using a prediction equation derived from appendicular lean soft tissue that incorporates both sex and age. Future studies using direct measurements of SMM, such as magnetic resonance imaging (37), are needed to confirm the independent role of skeletal muscle in determining RMR.

Consistent with a previous study (35), our findings demonstrated a significant positive correlation between FFM, SMM, and RMR. The correlation coefficients for RMR and FFM were generally consistent with prior studies (33, 36), while the correlation coefficient for SMM was higher than that of the nonathlete group (44). Furthermore, we conducted separate multiple regression analyses for male and female athletes to analyze the contribution of predictive variables. To examine a more homogeneous sample, a subgroup analysis including only long-distance runners from the present cohort was performed. In multiple regression analysis with sex entered as a dummy variable, Model 1 including FM and FFM identified only FFM as a significant variable (R^2^ = 0.750). In Model 2 including FM, SMM, and residual, only SMM remained a significant variable (R^2^ = 0.766). These results were consistent with those obtained from the analysis including all sports disciplines. Among nonathletes, both FFM and FM were identified as significant variables (45). In contrast, in both male and female athletes in the same ethnic group, only FFM was selected as a significant variable in Model 1, whereas FM was not. These findings are consistent with those of previous studies on athletes (16, 31, 46), suggesting that FFM alone contributes to RMR in athletes. Furthermore, in Model 2, SMM was the only significant variable for both male and female athletes. This is likely due to the differences in body composition between athletes and nonathletes. A previous study (47) reported that exercise-induced increases in FFM were significantly correlated with variations in RMR after strength-training. A report indicated that gains or losses in SMM are key variables in the fluctuating RMR observed during weight changes in nonathletes (48). Although FM was not identified as a significant variable in the female athletes, the *p*-values approached the significance threshold. Therefore, the relatively small female sample size and greater metabolic variability among female athletes may have reduced statistical power and increased the possibility of a type II error. In addition, the wider confidence intervals observed in female athletes suggest less precise estimation of regression coefficients compared with male athletes. Consequently, the potential contribution of FM to RMR in female athletes cannot be completely excluded. It should also be noted that the body composition variables included in the present analyses differed in their methodological basis. FM and FFM were directly derived from DXA measurements, whereas SMM was estimated using a prediction equation. In addition, residual mass was not directly measured but calculated by subtraction after accounting for FM, BM, and SMM. Thus, the residual component should not be interpreted as a direct measure of metabolically active organs. Even though SMM was identified as a significant variable in the regression models, these findings should be interpreted cautiously because both SMM and residual represent estimated rather than directly quantified physiological compartments.

One of the most important findings of the present study was the substantial sex difference in the explanatory power of the regression models. Although FFM and SMM were identified as significant variables of RMR in both male and female athletes, the coefficients of determination were markedly lower in female athletes than in male athletes. Specifically, body composition variables explained approximately 80% of the variance in RMR in male athletes but only approximately 40% in female athletes. This observation is similar to reports indicating that in male athletes, FFM accounts for 70% (48) of the RMR, whereas in female athletes, FFM accounts for 29% (14) and 28% (16) of the RMR. Furthermore, some studies have suggested that FFM explains 68% of RMR variation in male Japanese athletes (36) and 55% in female Japanese athletes (33). These findings suggest that RMR in female athletes may be influenced by a broader range of physiological factors beyond body composition alone. Previous studies have also reported lower predictive accuracy of FFM-based RMR equations in female athletes compared with male athletes (15), indicating that the relationship between metabolically active tissue and RMR may be more complex in women. Potential contributing variables include differences in fat distribution, endocrine status, menstrual-related hormonal variability, energy availability, and metabolic adaptations associated with chronic training. Although not a primary variable, metabolic activity involves fats other than FFM, and this proportion is expected to be higher in female athletes than in male athletes. As FM was selected as a predictive variable for RMR in nonathletes with higher body fat percentages than athletes, it is conceivable that female athletes with higher body fat percentages than male athletes may exhibit similar trends to nonathletes. Additionally, previous research has reported that prediction bias in RMR estimation increases in women with lower body fat percentages (15). Because the female athletes in the present study were relatively low body fat percentage compared to the previous studies may have (14, 49), their low body fat percentage may have contributed to the lower coefficient of determination observed in the regression model. Furthermore, differences in T_3_ hormone concentrations, which have been reported to be related to metabolism (50), may also contribute to RMR. T_3_ has been shown to explain an additional 2%–8% of RMR variance beyond body composition (36, 51, 52), and subtle hormonal variability—even within the normal range—may contribute to residual unexplained variance. Although T_3_ levels were within the normal range in both male and female athletes, values were significantly higher in male athletes. The exploratory models including T_3_ provide additional insight into the potential contribution of endocrine factors to RMR. In male athletes, T_3_ remained a significant variable after adjustment for body composition, suggesting that thyroid hormone status may contribute modestly to individual variation in RMR, even within the normal physiological range. However, the increase in the coefficient of determination after adding T_3_ was small, indicating that body composition, particularly FFM or SMM, remained the dominant significant variable. In contrast, T_3_ was not an independent variable in female athletes after adjustment for body composition variables. Previous research in female athletes identified T_3_ as a significant variable in addition to FFM, with an additional contribution of 2.3% to the explained variance in RMR (51). In the present study, adding T_3_ to Model 1 increased the coefficient of determination by 1.8% in female athletes. Although T_3_ did not reach statistical significance, the magnitude of this additional contribution was broadly comparable with that reported previously. Therefore, the discrepancy between studies may partly reflect the relatively smaller sample size in the present study and the resulting limited statistical power to detect modest endocrine contributions. These findings may reflect subtle differences in metabolic and endocrine status between sexes. In female athletes, the determinants of RMR may be more complex and less strongly explained by body composition than in male athletes, suggesting that multiple physiological variables may interact to influence metabolic rate. Nevertheless, caution is warranted in interpreting these findings, as energy status and dietary intakes were not systematically assessed in this study. Further longitudinal investigations are warranted to elucidate the effects of hormonal changes on RMR. Taken together, the findings from the multiple regression analyses may also have important practical implications for estimating RMR in female athletes. Prediction equations primarily based on FFM may provide less precise estimates in female athletes than in male athletes, and reliance on body composition variables could underestimate inter-individual metabolic variability in female athletes. These findings highlight the need for more comprehensive prediction models that incorporate not only body composition but also endocrine and metabolic variables to improve the assessment of energy requirements in female athletes.

The findings of this study may have practical implications for sports nutritionists, coaches, and practitioners involved in monitoring energy availability in athletes. Although FFM-based approaches appear useful for estimating RMR in both sexes, the substantially lower explained variance observed in female athletes suggests that body composition alone may not fully capture the determinants of RMR in women. Thus, caution may be warranted when applying generalized prediction equations to female athletes, particularly those with relatively low body fat percentages or potential fluctuations in energy status and endocrine status. Accordingly, the present findings suggest that more individualized approaches incorporating not only body composition variables but also physiological and endocrine variables may be necessary for accurately evaluating RMR and energy requirements in female athletes.

This study had certain limitations. First, because of the cross-sectional design, causal relationships between body composition variables and RMR could not be determined. Second, sex hormone or catecholamine levels, which have been shown to influence RMR, were not evaluated. Additionally, female athletes were evaluated during the early follicular phase based on the doctor's judgment; however, ovulation was not identified. Furthermore, energy status was not systematically evaluated, limiting interpretation of the metabolic significance of hormonal differences observed between sexes. Third, although participants were instructed to avoid strenuous exercise before testing, complete standardization of prior training load was not always possible. Residual effects of recent exercise or recovery status may therefore have contributed to individual variability in RMR. Fourth, SMM was not directly measured but estimated using a prediction equation based on appendicular lean soft tissue, sex, and age. In addition, residual mass was calculated indirectly by subtraction rather than directly measured. Accordingly, interpretation of the contribution of specific tissues to RMR should be made cautiously. Fifth, seven female athletes with implausibly low RMR values were excluded from the primary analyses. Although these values were considered likely to reflect measurement-related abnormalities based on laboratory reference data, the possibility that they reflected physiological variation related to low energy status cannot be completely excluded. Finally, the included sports were heterogeneous and consisted of endurance, strength, and team sport athletes with different training characteristics, body composition, and metabolic adaptations. Although an additional subgroup analysis restricted to long-distance runners yielded similar results, sport-specific characteristics may still have influenced the findings. In addition, because the participants were Japanese national-level athletes classified as Tier 3, the generalizability of the findings to other ethnicities, competitive levels, or nonathlete populations may be limited. Future studies with larger sample sizes should examine homogeneous sport groups to better clarify the sex differences of RMR in athletes.

## 5 Conclusions

FFM and SMM were significantly associated with RMR in both Japanese male and female athletes, but their contributions differed between sexes. Sex differences in absolute RMR may be partly attributable to differences in body composition and absolute body size, while sex-related differences in RMR should be interpreted cautiously. Although sex differences in absolute RMR were attenuated after normalization to FFM, such normalization may not fully account for physiological differences in body composition and metabolism between male and female athletes. The predictive models appeared to be more robust in male athletes than in female athletes, suggesting that additional physiological or endocrine factors may contribute to RMR variability in female athletes.

## Data Availability

The datasets presented in this article are not readily available because of privacy reasons. Requests to access the datasets should be directed to Motoko Taguchi, mtaguchi@waseda.jp.
